# Novel and Highly Efficient Regioselective Route to Helicid Esters by Lipozyme TLL

**DOI:** 10.1371/journal.pone.0080715

**Published:** 2013-11-22

**Authors:** Rongling Yang, Xiangjie Zhao, Xueming Liu

**Affiliations:** 1 Sericulture and Agri-Food Research Institute, Guangdong Academy of Agricultural Sciences, Guangzhou, China; 2 College of Light Industry and Food Sciences, South China University of Technology, Guangzhou, China; Oak Ridge National Laboratory, United States of America

## Abstract

Highly regioselective acylation of helicid with fatty acid vinyl esters catalyzed by the lipase from *Thermomyces lanuginosus* has been successfully performed for the first time. For the enzymatic caproylation of helicid, under the optimal conditions, initial reaction rate was 33.2 mM/h, and substrate conversion and regioselectivity were greater than 99%. In addition, the acyl recognition of the enzyme in the regioselective acylation of helicid was investigated. The results showed that although 6’-*O*-acyl derivatives of helicid were exclusively obtained with all the tested acyl donors, the enzymatic reaction rate varied widely with different acyl donors, presumably owing to their different interactions with the active site of the lipase. It is also interesting that the different configuration of only one hydroxyl group at C-3 in helicid couldn’t affect the lipase-catalyzed esterification and helicid has the same regioselectivity as that of D-glucose and arbutin.

## Introduction

Helicid, namely *p*-formylphenyl *β*-D-allopyranoside, was originally isolated as one of the main active constituents from *Helicid nilgrinica* Bedd, a traditional Chinese herb. It has been used clinically as antalgic and hypnotic for a long time in China. Some studies also found that helicid could inhibit cholinesterase or tyrosinase activities [1], [2]. However, as a therapeutic agent, helicid suffers from low oral bioavailability due to its poor cell membrane penetration and its activity could be enhanced significantly by introducing an appropriate lipophilic group into its structure.

Recently, it was reported that ester derivatives of helicid had higher inhibitory activities toward cholinesterase and mushroom tyrosinase, presumably due to their increased solubility in oil-based systems and improved membrane penetration [1], [2]. For example, when acetylthiocholine and butylthiocholine were used as the substrate, helicid acetic ester caused 50% inhibition of cholinesterase at a concentration of less than 10 mM, compared to a concentration of free helicid of 500 mM that was required to have the same inhibitory effect [1].

Helicid has several hydroxyls with similar chemical reactivity and so it is extremely difficult to acylate a single specific hydroxyl in unprotected helicid directly via conventional chemical approaches, unless time-consuming protection–deprotection steps are employed. Fortunately, enzymatic regioselective acylation is a useful alternative to classical chemical methods, and offers high selectivity, simplicity and environmental friendliness [3], [4], [5], [6], [7]. We previously obtained several fatty acid esters of arbutin catalyzed by immobilized lipase from *Penicillium expansum*, with high conversion and excellent 6’-regioselectivity [8], [9]. However, as arbutin’s analogue, there have been few reports on the enzymatic acylation of helicid up to now. It is also interesting whether the different configuration of only one hydroxyl group at C-3 in helicid may affect the lipase-catalyzed esterification and whether the same regioselectivity as that of D-glucose and arbutin are observed.

**Figure 1:**
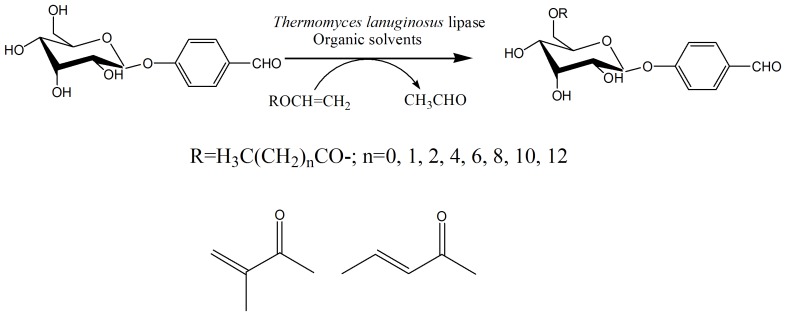
Enzymatic regioselective acylation of helicid.

Lipozyme TLL, an immobilized lipase from *Thermomyces lanuginosus*, is a low-cost lipase that has important industrial applications in the synthesis of sugar esters [10] and oil esters [11], resolution of chiral alcohol [12], preparation of biodiesel [13] and acylation of nucleosides [5], [6]. Here we have investigated the potential of lipozyme TLL for regioselective acylation of helicid, and have obtained several fatty acid esters of helicid with high conversion and excellent 6’-regioselectivity (Figure 1).

## Materials and Methods

### Biological and Chemical Materials

*Candida antarctica* lipase B (Novozym 435, CAL-B), *Thermomyces lanuginosus* lipase (Lipozyme TL IM, TLL), *Rhizomucor miehei* lipase (Lipozyme RM IM, RML) were purchased from Novozymes Co., Ltd., China. *Candida rugosa* lipase (powder, CRL) was from Meito SangyoCo., Japan. *Penicillium roqueforti* lipase (PRL, Lipase R) and *Penicillium camemberti* lipase (PCL, Lipase G) are powder from Amano Enzyme Inc., Japan. Helicid and vinyl esters used as the acyl donors were purchased from TCI and Alfa Aesar. Other chemicals were from commercial sources and were of the highest purity available.

### Assaying of Enzyme Esterification Activity

The enzyme esterification activity was determined according to the method [14]. The specific activities of CAL-B, TLL, RML, CRL, PCL and PRL were 2.5, 0.21, 0.27, 0.68, 0.13 and 2.71 U/mg, respectively.

### General Procedure for Enzymatic Acylation of Helicid

In a typical experiment, helicid (0.02 mmol), Lipozyme TLL and fatty acid vinyl ester were added into 2 ml anhydrous THF and the mixture was incubated at a predetermined temperature in an orbital air-bath shaker (200 rpm). Aliquots were withdrawn at specified time intervals from the reaction mixture, and then diluted 50-fold with corresponding mobile phase prior to HPLC analysis. Regioselectivity was defined as the molar ratio of the desired product to the total amount of ester products formed. All data are averages of experiments performed in triplicate. No chemical acylation of helicid was detectable in controls from which the lipase preparation was omitted.

### Operational Stability

Anhydrous THF (2 ml), helicid (0.02 mmol), vinyl hexanoate (0.15 mmol) and enzyme (20 U) were incubated at 200 rpm and 45°C for 1.5 h. Then, the enzyme was separated by filtration, thoroughly washed with reaction medium and added into fresh reaction mixture to catalyze the acylation of helicid with a new aliquot of the same amount of vinyl hexanoate. The process was repeated to obtain the operational stability of the enzyme after up to 11 cycles of reaction.

### HPLC Analysis

The reaction mixture was analyzed by RP-HPLC on a 4.6 mm×250 mm (5 µm) Zorbax SB-C18 column (Agilent Technologies Industries Co., Ltd., USA) using an Agilent G1311A pump and a UV detector at 270 nm. The mobile phase is a mixture of water and methanol at 1.0 ml/min. The volumetric ratio of water to methanol and the retention times for helicid and its 6’-*O*-monoester were 60/40, 3.210 and 6.808 min (acetylation), 60/40, 3.198 and 10.442 min (propionylation), 40/60, 2.657 and 4.578 min (butyrylation), 20/80, 2.511 and 3.921 min (hexanoylation), 20/80, 2.509 and 4.797 min (caproylation), 20/80, 2.512 and 7.704 min (decanoylation), 10/90, 2.409 and 5.189 min (lauroylation), 10/90, 2.413 and 7.498 min (myristoylation), respectively. A gradient elution with water/methanol of 40/60 (v/v) from 0 to 3 min, and then water/methanol of 20/80 (v/v) at 5.0 min was used for crotonylation and methacryloylation. The retention times for helicid and its 6’-*O*-monoester were 2.621, 4.029 (crotonylation) and 4.414 min (methacryloylation), respectively.

### Scale-up Synthesis and Purification of the Esters and Structure Determination

The reaction was initiated by adding 200 U Lipozyme TLL to 20 ml anhydrous THF containing 0.2 mmol helicid and 1.5 mmol acyl donor at 200 rpm and 45°C. After the reaction, the enzyme was removed by filtration and the solvent was evaporated under vacuum. The residue was then purified through flash column chromatography using ethyl acetate/petroleum ether as the mobile phase. The products were exclusively helicid 6’-esters as characterized by ^13^C NMR and ^1^H NMR (Bruker DRX-400 NMR Spectrometer, Bruker Co., Germany) at 100 MHz and 400 MHz, respectively, with DMSO-*d*_6_ being the solvent. Results from the NMR spectroscopy are given in Figure S1. Mass spectra were recorded on LCQ Deca Xp (Thermo Finnigan) using ESI mode with ion spray voltage 3000 V. The sheath gas arbitrary flow was set at 15 arb. The capillary temperature and voltage were 250°C and 18 V, respectively. Results from the mass spectra are given in Figure S3. In addition, the HPLC chromatograms of the helicid ester derivatives are provided in Figure S2.

### Helicid

^1^H NMR (400 MHz, DMSO-*d_6_*): *δ* 3.42–3.50 (m, 3, H_2'_+ H_3'_+ H_4'_), 3.67–3.72 (m, 1, H_5'_), 3.74–3.78 (apparent d, 1, *J* = 3.2 Hz, H_6'_), 3.96 (apparent d, 1, *J* = 3.2 Hz, H_6'_), 4.52 (t, 1, *J* = 5.7, 6.6 Hz, OH_6'_), 4.71 (d, 1, *J* = 7.4 Hz, H_1'_), 5.01 (d, 1, *J* = 3.7 Hz, OH_4'_), 5.15 (d, 1, *J* = 6.8 Hz, OH_3'_), 5.27 (d, 1, *J* = 7.9 Hz, OH_2'_), 7.19 (d, 2, *J* = 8.7 Hz, H_2_+ H_6_), 7.87 (d, 2, *J* = 8.7 Hz, H_3_+ H_5_), 9.89 (s, 1, OH_7_). ^13^C NMR (100 MHz, DMSO-*d_6_*): *δ* 60.86 (C_6'_), 66.93 (C_4'_), 70.18 (C_2'_), 71.45 (C_3'_), 74.79 (C_5'_), 98.08 (C_1'_), 116.39 (C_2_+ C_6_), 130.45 (C_4_), 131.65 (C_3_+ C_5_), 162.38 (C_1_), 191.45 (C_7_).

### Helicid 6’-acetate

^1^H NMR: *δ* ppm 2.01(s, 3, H_2''_), 3.46–3.55 (m, 2, H_2'_+ H_3'_), 4.01 (apparent dd, 2, *J* = 16.3, 5.6 Hz, H_4'_+ H_5'_), 4.10 (dd, 1, *J* = 11.7, 6.6 Hz, H_6'_), 4.27–4.31 (m, 1, H_6'_), 4.98 (d, 1, *J* = 7.4 Hz, H_1'_), 5.15 (d, 1, *J* = 3.7 Hz, OH_4'_), 5.28 (dd, 2, *J* = 7.9 Hz, OH_2'_+OH_3'_), 7.19 (d, 2, *J* = 8.7 Hz, H_2_+ H_6_), 7.89 (d, 2, *J* = 8.7 Hz, H_3_+ H_5_), 9.90 (s, 1, OH_7_). ^13^C NMR: δ ppm 20.62 (C_2''_), 63.59 (C_6'_), 67.13 (C_4'_), 69.97 (C_2'_), 71.28 (C_3'_), 71.44 (C_5'_), 97.85 (C_1'_), 116.29 (C_2_+ C_6_), 130.52 (C_4_), 131.59 (C_3_+ C_5_), 162.06 (C_1_), 170.24 (C_1''_), 191.42 (C_7_). The isolated yield was 76%.

### Helicid 6’-propionate

^1^H NMR: δ ppm 1.01(t, 3, *J* = 7.5 Hz, H_3''_),2.31(qd, 2, *J* = 7.6, 2.0 Hz, H_2''_), 3.45–3.55 (m 2, H_2'_+ H_3'_), 3.97–4.05 (m 2, H_4'_+ H_5'_), 4.10 (dd, 1, *J* = 11.7, 7.0 Hz, H_6'_), 4.32 (dd, 1, *J* = 11.9, 2.0 Hz, H_6'_), 4.98 (d, 1, *J* = 7.4 Hz, H_1'_), 5.15 (d, 1, *J* = 3.8 Hz, OH_4'_), 5.26 (dd, 2, *J* = 12.7, 7.3 Hz, OH_2'_+OH_3'_), 7.19 (d, 2, *J* = 15.9 Hz, H_2_+ H_6_), 7.88 (d, 2, *J* = 7.6 Hz, H_3_+ H_5_), 9.90 (s, 1, OH_7_). ^13^C NMR: δ ppm9.40 (C_3''_), 27.27 (C_2''_), 64.08 (C_6'_), 67.71 (C_4'_), 70.47 (C_2'_), 71.79 (C_5'_), 72.05 (C_3'_), 98.34 (C_1'_), 116.79 (C_2_+ C_6_), 131.02 (C_4_), 132.05 (C_3_+ C_5_), 162.57 (C_1_), 173.93 (C_1''_), 191.91 (C_7_). The isolated yield was 80%.

### Helicid 6’-butyrate

^1^H NMR: δ ppm 0.85 (t, 3, *J* = 7.5, H_4''_), 1.51 (q, 2, *J* = 7.3 Hz, H_3''_), 2.27 (td, 2, *J* = 7.2, 1.4 Hz, H_2''_), 3.42–3.53 (m, 2, H_2'_+H_3'_), 3.95–4.04 (m, 2, H_4'_+H_5'_), 4.09 (dd, 1, *J* = 11.7, 7.0 Hz, H_6'_), 4.31 (apparent dd, 1, *J* = 11.9, 2.0 Hz, H_6'_), 4.97 (d, 1, *J* = 7.4 Hz, H_1'_), 5.14 (d, 1, *J* = 3.8 Hz, OH_4'_), 5.25 (dd, 2, *J* = 12.7, 7.3 Hz, OH_2'_+ OH_3'_), 7.18 (d, 2, *J* = 12.0 Hz, H_2_+ H_6_), 7.88 (d, 2, *J* = 8.0 Hz, H_3_+ H_5_), 9.90 (s, 1, OH_7_). ^13^C NMR: δ ppm 13.35 (C_4''_), 17.87 (C_3''_), 35.32 (C_2''_), 63.52 (C_6'_), 67.24 (C_4'_), 69.95 (C_2'_), 71.28 (C_5'_), 71.55 (C_3'_), 97.80 (C_1'_), 116.30 (C_2_+ C_6_), 130.52 (C_4_), 131.54 (C_3_+ C_5_), 162.07 (C_1_), 172.58 (C_1''_), 191.43 (C_7_). The isolated yield was 78%.

### Helicid 6’-hexanoate

^1^H NMR: δ ppm 0.80 (apparent t, 3, H_6''_), 1.16–1.24 (m, 4, H_4''_+H_5''_), 1.45–1.54 (m, 2, H_3''_), 2.28 (td, 1, *J* = 7.3, 1.3 Hz, H_2''_), 3.44–3.55 (m, 2, H_2'_+H_3'_), 3.94–4.04 (m, 2, H_4'_+H_5'_), 4.10 (apparent dd, 1, *J* = 11.7, 7.1 Hz, H_6'_), 4.32 (apparent d, 1, *J* = 8.0 Hz, H_6'_), 4.97 (d, 1, *J* = 7.4 Hz, H_1'_), 5.15 (d, 1, *J* = 3.8 Hz, OH_4'_), 5.26 (dd, 2, *J* = 14.7, 7.3 Hz, OH_2'_+ OH_3'_), 7.18 (apparent d, 2, *J* = 8.0 Hz, H_2_+ H_6_), 7.88 (apparent d, 2, *J* = 8.0 Hz, H_3_+ H_5_), 9.90 (s, 1, OH_7_). ^13^C NMR: δ ppm 13.69 (C_6''_), 21.71 (C_5''_), 24.06 (C_4''_), 30.58 (C_3''_), 33.42 (C_2''_), 63.54 (C_6'_), 67.29 (C_4'_), 69.97 (C_2'_), 71.28 (C_5'_), 71.54 (C_3'_), 97.78 (C_1'_), 116.27 (C_2_+ C_6_), 130.50 (C_4_), 131.52 (C_3_+ C_5_), 162.07 (C_1_), 172.70 (C_1''_), 191.31 (C_7_). The isolated yield was 82%.

### Helicid 6’-caprylate

^1^H NMR: δ ppm 0.82 (t, 3, *J* = 6.7 Hz, H_8''_), 1.15–1.23 (m, 8, H_4''_+ H_5''_+ H_6''_+ H_7''_), 1.44–1.56 (m, 2, H_3''_), 2.28 (t, 2, *J* = 7.4 Hz, H_2''_), 3.43–3.48 (m, 1, H_3'_), 3.52 (td, 1, *J* = 7.2, 2.7 Hz, H_2'_), 3.96–4.03 (m, 2, H_4'_+ H_5'_), 4.06–4.11 (m, 1, H_6'_), 4.31 (apparent dd, 1, *J* = 11.8, 2.0 Hz, H_6'_), 4.96 (d, 1, *J* = 7.2 Hz, H_1'_), 5.13 (apparent dd, 1, *J* = 8.8, 3.8 Hz, OH_4'_), 5.20–5.30 (m, 2, OH_2'_+ OH_3'_), 7.16–7.19 (m, 2, H_2_+ H_6_), 7.86–7.89 (m, 2, H_3_+ H_5_), 9.90 (s, 1, OH_7_).^ 13^C NMR: δ ppm 13.85 (C_8''_), 21.96 (C_7''_), 24.40 (C_3''_), 28.30 (C_5''_), 28.37 (C_4''_), 31.05 (C_6''_), 33.49 (C_2''_), 63.56 (C_6'_), 67.30 (C_4'_), 69.97 (C_2'_), 71.29 (C_5'_), 71.54 (C_3'_), 97.82 (C_1'_), 116.27 (C_2_+ C_6_), 130.50 (C_4_), 131.51 (C_3_+ C_5_), 162.09 (C_1_), 172.70 (C_1''_), 191.27 (C_7_). The isolated yield was 85%.

### Helicid 6’-decanoate

^1^H NMR: δ ppm 0.83 (t, 3, *J* = 6.8 Hz, H_12''_), 1.18–1.21 (m, 12, H_4''_+ H_5''_+ H_6''_+ H_7''_+ H_8''_+ H_9''_), 1.47 (p, 2, *J* = 7.1 Hz, H_3''_), 2.27 (t, 2, *J* = 7.4 Hz, H_2''_), 3.51–3.55 (m, 2, H_2'_+ H_3'_), 3.98–4.04 (m, 2, H_4'_+ H_5'_), 4.10 (dd, 1, *J* = 11.7, 7.1 Hz, H_6'_), 4.32 (d, 1, *J* = 11.6 Hz, H_6'_), 4.97 (d, 1, *J* = 7.4 Hz, H_1'_), 5.15 (apparent d, 1, *J* = 3.8 Hz, OH_4'_), 5.26 (t, 2, *J* = 7.2 Hz, OH_2'_+ OH_3'_), 7.17 (d, 2, *J* = 8.4 Hz, H_2_+ H_6_), 7.87 (d, 2, *J* = 8.3 Hz, H_3_+ H_5_), 9.89 (s, 1, OH_7_). ^13^C NMR: δ ppm 13.83 (C_10''_), 22.03 (C_9''_), 24.40 (C_3''_), 28.43 (C_4''_), 28.60 (C_7''_), 28.67 (C_6''_), 28.80 (C_5''_), 31.22 (C_8''_), 33.49 (C_2''_), 63.58 (C_6'_), 67.32 (C_4'_), 69.98 (C_2'_), 71.26 (C_5'_), 71.54 (C_3'_), 97.84 (C_1'_), 116.26 (C_2_+ C_6_), 130.49 (C_4_), 131.47 (C_3_+ C_5_), 162.10 (C_1_), 172.67 (C_1''_), 191.16 (C_7_). The isolated yield was 89%.

### Helicid 6’-laurate

^1^H NMR: δ ppm 0.85 (apparent t, 3, *J* = 6.6 Hz, H_12''_), 1.19–1.24 (m, 16, H_4''_+ H_5''_+ H_6''_+ H_7''_+ H_8''_+ H_9''_+ H_10''_ +H_11''_), 1.48 (apparent t, 2, *J* = 7.3 Hz, H_3''_), 2.28 (t, 2, *J* = 7.5 Hz, H_2''_), 3.42–3.53 (m, 2, H_2'_+ H_3'_), 3.95–4.02 (m, 2, H_4'_+ H_5'_), 4.08 (dd, 1, *J* = 11.7, 7.1 Hz, H_6'_), 4.30 (d, 1, *J* = 11.4 Hz, H_6'_), 4.96 (d, 1, *J* = 7.4 Hz, H_1'_), 5.14 (d, 1, *J* = 3.8 Hz, OH_4'_), 5.24 (apparent dd, 2, *J* = 10.3, 7.4 Hz, OH_2'_+ OH_3'_), 7.17 (d, 2, *J* = 8.4 Hz, H_2_+ H_6_), 7.87 (d, 2, *J* = 8.3 Hz, H_3_+ H_5_), 9.90 (s, 1, OH_7_).^ 13^C NMR: δ ppm 13.91 (C_12''_), 22.05 (C_11''_), 24.40 (C_3''_), 28.42 (C_4''_), 28.66 (C_5''_+C_9''_), 28.83 (C_7''_), 28.94 (C_6''_+C_8''_), 31.25 (C_10''_), 33.48 (C_2''_), 63.56 (C_6'_), 67.29 (C_4'_), 69.96 (C_2'_), 71.28 (C_5'_), 71.53 (C_3'_), 97.83 (C_1'_), 116.27 (C_2_+ C_6_), 130.50 (C_4_), 131.52 (C_3_+ C_5_), 162.09 (C_1_), 172.70 (C_1''_), 191.27 (C_7_). The isolated yield was 87%.

### Helicid 6’-myristate

^1^H NMR: δ ppm 0.86 (t, 3, *J* = 6.6 Hz, H_14''_), 1.21 (apparent d, 20, *J* = 15.7 Hz, H_4''_+ H_5''_+ H_6''_+ H_7''_+ H_8''_+ H_9''_+ H_10''_ +H_11''_+ H_12''_+ H_13''_), 1.48 (apparent p, 2, *J* = 7.1 Hz, H_3''_), 2.28 (t, 2, *J* = 7.3 Hz, H_2''_), 3.41–3.53 (m, 2, H_2'_+ H_3'_), 3.95–4.02 (m, 2, H_4'_+ H_5'_), 4.08 (dd, 1, *J* = 11.7, 7.1 Hz, H_6'_), 4.30 (apparent dd, 1, *J* = 11.8, 2.0 Hz, H_6'_), 4.96 (d, 1, *J* = 7.4 Hz, H_1'_), 5.15 (apparent d, 1, *J* = 3.8 Hz, OH_4'_), 5.24 (t, 2, *J* = 8.3, Hz, OH_2'_+ OH_3'_), 7.17 (apparent d, 2, *J* = 8.0 Hz, H_2_+ H_6_), 7.88 (apparent d, 2, *J* = 8.0 Hz, H_3_+ H_5_), 9.90 (s, 1, OH_7_). ^13^C NMR: δ ppm 13.92 (C_14''_), 22.06 (C_13''_), 24.40 (C_3''_), 28.42 (C_4''_), 28.66 (C_5''_+C_11''_), 28.83 (C_6''_), 28.92 (C_7''_), 28.97 (C_9''_+C_8''_), 28.99 (C_10''_), 31.26 (C_12''_), 33.49 (C_2''_), 63.56 (C_6'_), 67.29 (C_4'_), 69.96 (C_2'_), 71.28 (C_5'_), 71.53 (C_3'_), 97.82 (C_1'_), 116.27 (C_2_+ C_6_), 130.49 (C_4_), 131.52 (C_3_+ C_5_), 162.09 (C_1_), 172.70 (C_1''_), 191.28 (C_7_). The isolated yield was 86%.

### Helicid 6’-methacrylate

^1^H NMR: δ ppm 1.88 (s, 3, H_4''_), 3.43–3.55 (m, 2, H_2'_+H_3'_), 3.97 (apparent d, 1, *J* = 3.2 Hz, H_4'_), 4.03–4.13 (m, 2, H_5_+H_6'_), 4.42 (d, 1, *J* = 10.0 Hz, H_6'_), 5.01 (d, 1, *J* = 7.4 Hz, H_1'_), 5.15 (d, 1, *J* = 6.3 Hz, OH_4'_), 5.25 (dd, 2, *J* = 13.5, 7.4 Hz, OH_2'_+ OH_3'_), 5.71 (s, 1, H_3''_), 6.07 (s, 1, H_3''_), 7.18 (d, 2, *J* = 8.7 Hz, H_2_+ H_6_), 7.84 (d, 2, *J* = 8.7 Hz, H_3_+ H_5_), 9.90 (s, 1, OH_7_). ^13^C NMR: δ ppm 17.93 (C_4''_), 64.29 (C_6'_), 67.37 (C_4'_), 69.97 (C_2'_), 71.34 (C_5'_), 71.55 (C_3'_), 97.83 (C_1'_), 116.30 (C_2_+ C_6_), 125.85 (C_3''_), 130.54 (C_4_), 131.50 (C_3_+ C_5_), 135.79 (C_2''_), 162.07 (C_1_), 166.35 (C_1''_), 191.46 (C_7_). The isolated yield was 75%.

### Helicid 6’-crotonate

^1^H NMR: δ ppm 1.86 (dd, 3, *J* = 6.9, 1.6 Hz, H_4''_), 3.47–3.53 (m, 2, H_2'_+H_3'_), 3.97 (s, 1, H_4'_), 4.01–4.06 (m, 1, H_5'_), 4.14 (dd, 1, *J* = 11.8, 6.9 Hz, H_6'_), 4.34 (dd, 1, *J* = 11.7, 1.7 Hz, H_6'_), 4.99 (apparent s, 1, H_1'_), 5.15 (s, 1, OH_4'_), 5.26 (d, 2, *J* = 8.0 Hz, OH_2'_+ OH_3'_), 5.90 (dd, 1, *J* = 15.5, 1.7 Hz, H_2''_), 6.90 (dq, 1, *J* = 13.8, 6.9 Hz, H_3''_), 7.17 (d, 2, *J* = 8.7 Hz, H_2_+ H_6_), 7.86 (d, 2, *J* = 8.8 Hz, H_3_+ H_5_), 9.90 (s, 1, OH_7_). ^13^C NMR: δ ppm 17.66 (C_4''_), 63.51 (C_6'_), 67.26 (C_4'_), 69.97 (C_2'_), 71.33 (C_5'_), 71.49 (C_3'_), 97.84 (C_1'_), 116.34 (C_2_+ C_6_), 122.13 (C_3''_), 130.50 (C_4_), 131.56 (C_3_+ C_5_), 145.34 (C_2''_), 162.04 (C_1_), 165.37 (C_1''_), 191.44 (C_7_). The isolated yield was 60%.

## Results and Discussion

### Screening the Biocatalyst

With the regioselective caproylation of helicid as a model reaction, three immobilized enzymes (CAL-B, TLL and RML) and three enzyme powders (PCL, PRL and CRL) were tested as the biocatalysts (Table 1). Among these lipases, lipozyme TLL showed the highest catalytic activities (11.9 mM/h), affording 98% conversion after 10 h, while the reaction catalyzed by lipase CAL-B and RML proceeded with low reaction rate and low conversion. Furthermore, no acylation products were detected in the reaction mixture by using the enzyme powders (PCL, PRL and CRL). The possible reason for no esterification activity is that the three lipase powders might be in the less active conformation, which is unfavorable for helicid of large size to enter into the active site, while water molecules of small size could readily enter into the active site and attack the acyl-enzyme intermediate.

**Table 1:** Regioselective caproylation of helicid catalyzed by various lipases.

Enzyme	V_0_ (mM/h)	Time (h)^a^	*C* (%)	6’-Regioselectivity (%)
CAL-B	5.1	14	53.2	>99
Lipozyme TLL	11.9	10	98	>99
RML	4.5	16	37.9	>99
PCL	n.d.	48	n.d.	n.d.
PRL	n.d.	48	n.d.	n.d.
CRL	n.d.	48	n.d.	n.d.

Reaction conditions: 0.02 mmol helicid, 0.1 mmol vinyl hexanoate, 10 µ lipase, 2 ml anhydrous THF, 40°C, 200 rpm.

a Reaction time when the maximum conversion was achieved.

n.d.: no detected.

Interestingly, all the lipases displayed absolute 6’-regioselectivities (>99%) in the caproylation of helicid. This is similar to the excellent selectivity toward the 6’-hydroxyl of the D-allose that was observed during acylation of D-allose catalysed by lipase from *Candida antarctica*, porcine pancreatic or *Burkholderia cepacia* [15]. Likewise, our group recently found that lipase from *Candida antarctica*, *Penicillium expansum*, *Pseudomonas cepacia* or *Thermomyces lanuginosus* exhibited excellent selectivity toward 6’-hydroxyl of the glucose moiety in the acylation of arbutin [9].

### Optimization of Enzymatic Caproylation of Helicid

With caproylation as a model reaction, the effects of several key variables were investigated in detail. As shown in Table 2, the reaction accelerated clearly with increasing enzyme dosage from 5 to 20 U (entries 1-4), and then no substantial variation occurred with further increasing amounts of enzyme.

**Table 2:** Optimization of enzymatic caproylation of helicid.

Entry	Enzyme dosage (U)	VB (eq.)	T (°C)	V_0_ (mM/h)	*C* (%)	6’-Regioselectivity (%)
1	5	5	40	3.4	97	>99
2	10	5	40	11.9	98	>99
3	15	5	40	16.2	>99	>99
4	20	5	40	24.4	>99	>99
5	25	5	40	25.1	>99	>99
6	30	5	40	26.2	>99	>99
7	20	1.5	40	6.9	58	>99
8	20	3	40	16.2	89	>99
9	20	7.5	40	30.3	>99	>99
10	20	10	40	31.4	>99	>99
11	20	15	40	32.2	>99	>99
12	20	7.5	35	26.7	>99	>99
14	20	7.5	45	33.2	>99	>99
15	20	7.5	50	33.5	>99	>99
16	20	7.5	55	33.1	>99	>99

Reactions conditions: 0.02 mmol helicid.

Parallel to enzymatic acylation of glycosides with vinyl esters, there exists a side reaction, the enzymatic hydrolysis of the acyl donors. As a result, an excess of the acyl donors is usually necessary in such reactions to ensure efficient enzymatic acylation. The molar ratio of vinyl hexanoate to helicid greatly influenced the initial acylation rate and the maximal conversion (Table 2, entries 4 and 7–11). A good initial reaction rate (30.3 mM/h) and high conversion (>99%) could be achieved with the molar ratio of vinyl hexanoate to helicid as 7.5 (Table 2, entry 9).

Generally, substrate molecules are more active at higher reaction temperatures. On the other hand, high temperature would induce the comformational changes of the enzyme, thus decreasing the enzyme activity. Hence, the effect of temperature on the reaction was examined. The reaction showed a broad temperature profile with an optimum at 45°C (entries 9 and 12–16). From these data, the optimum conditions of enzyme dosage, molar ratio of vinyl hexanoate to helicid and reaction temperature were 20 U, 7.5 and 45°C, respectively, and the regioselectivity of the reaction remained excellent under all conditions tested.

### Time Course of Enzymatic Reaction and Operational Stability

**Figure 2:**
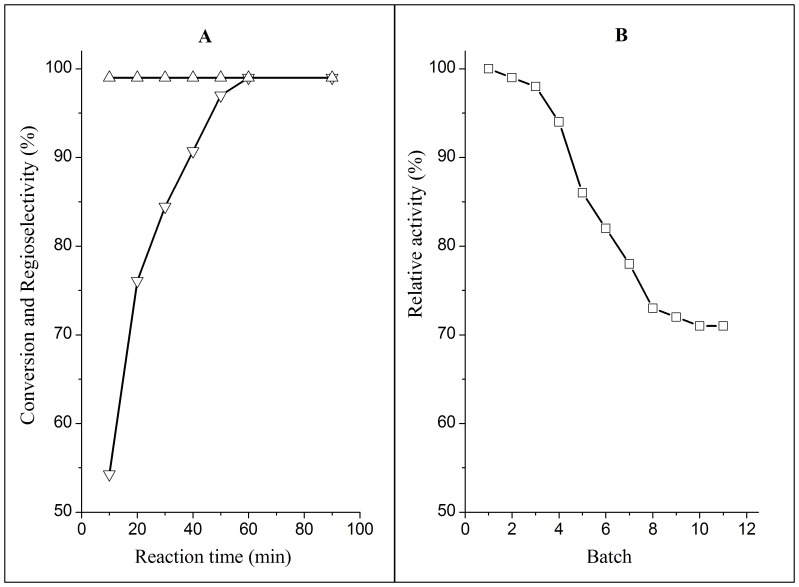
Time course of enzymatic caproylation and operational stability of *Thermomyces lanuginosus* lipase. Reactions conditions: 0.02 mmol helicid, 0.15 mmol vinyl hexanoate, 20 U *Thermomyces lanuginosus* lipase, 2 ml anhydrous THF, 45°C, 200 rpm. Symbols: (▽) the conversion, (△) the regioselectivity, (□) the relative activity.

To gain a deeper insight into the enzymatic progress, the time course of caproylation of helicid catalyzed by lipase TLL was followed under the optimum conditions described above. Substrate conversion increased rapidly with reaction time, and reached its maximum at 1.5h (Figure 2A). The lipase TLL showed the higher operational stability with 28% loss in activity after 8 cycles of the reaction (Figure 2B).

### Regioselective Acylation of Helicid with Various Acyl Donors

The acylation of helicid with various fatty acid vinyl esters catalyzed by lipase TLL was investigated in anhydrous THF (Table 3). Interestingly, in all cases lipase TLL displayed almost absolute 6’-regioselectivity (>99%), since only the 6’-ester of helicid could be detected by NMR and HPLC, which is similar to the acylation of sucrose, rutin, esculin, isoquercitrin and arbutin with a notable selectivity for 6’-hydroxyl of the glucose moiety [8], [9], [16], [17], [18]. This regioselectivity may occur because the less-hindered primary hydroxyl of the sugar moiety may more easily enter into the active site of the lipase to attack the acyl-enzyme intermediate than the other more hindered hydroxyl groups, thus resulting in the preferential formation of 6’-esters.

**Table 3:** Enzymatic synthesis of various esters of helicid.

Entry	Acyl donor	V_0_ (mM/h)	Time (h)^a^	*C* (%)	6’-Regioselectivity (%)
1	Vinyl acetate (C2)	24.3	4	>99	>99
2	Vinyl propionate (C3)	27.2	3	>99	>99
3	Vinyl butyrate (C4)	31.1	1.5	>99	>99
4	Vinyl hexanoate (C6)	33.2	1.5	>99	>99
5	Vinyl caprylate (C8)	38.3	1.0	>99	>99
6	Vinyl decanoate (C10)	37.2	1	>99	>99
7	Vinyl laurate (C12)	27.5	1.5	>99	>99
8	Vinyl myristate (C14)	20.8	2	>99	>99
9	Vinyl methacrylate (C4)	7.3	6	89	>99
10	Vinyl crotonate (C4)	0.9	23	92	>99

Reaction conditions: 0.02 mmol helicid, 0.15 mmol fatty acid vinyl ester, 20 µ lipase, 2 ml anhydrous THF, 45°C, 200 rpm.

a Reaction time when the maximum conversion was achieved.

As shown in Table 2, the initial reaction rate increased with the elongation of chain length of vinyl esters from C2 to C8 (Table 3, entries 1–5), perhaps because medium chain-length acyl groups can form stronger interactions with the hydrophobic acyl binding site of the enzyme than shorter-chain acyl groups [19], [20]. However, the initial reaction rate decreased with the elongation of chain length from C10 to C14 (Table 3, entries 6–8), presumably due to the larger steric hindrance of the longer chain acyl donors. This is similar to the results obtained in the acylation of nucleosides with the same lipase [21].

When there was a conjugated C–C double bond adjacent to the carbonyl moiety in the acyl group, the reaction rate decreased substantially (Table 3, entries 9, 10). Initial crotonylation and methacrylation rates were 0.9 and 7.3 mM/h, respectively, which were much lower than that of the butanoylation (31.1 mM/h, entry 3). This effect might be attributed to the resonance effect of the conjugate double bond [22]. Surprisingly, although vinyl crotonate is less hindered than vinyl methacrylate due to the presence of *α*-methyl group in the latter, the reaction rate with vinyl methacrylate was greater than that with vinyl crotonate. Recently, we obtained similar results in enzymatic acylation of arbutin: a conversion of 99% at 20 h was afforded with vinyl methacrylate as the acyl donor, in contract to the same conversion of 99% at 72 h with vinyl crotonate [9].

## Conclusions

In conclusion, various 6’-ester derivatives of helicid could be synthesized via lipase-mediated transesterification with good conversions and excellent regioselectivities. The structure of the acyl donors brings a significant impact on the catalytic performance of lipozyme TLL. These findings will undoubtedly enrich the fundamentals of enzymology. Furthermore, the enzymatic process is highly regioselective, simple, environmentally friendly and mild as compared with the traditional chemical procedures.

## Supporting Information

Figure S1 NMR spectra of 6’-ester derivatives of helicid.
(DOC)

Figure S2 HPLC Chromatograms of 6’-ester derivatives of helicid.
(DOC)

Figure S3 MS spectra of 6’-ester derivatives of helicid.
(DOC)
