# Supplementary material for: Novel and Highly Efficient Regioselective Route to Helicid Esters by Lipozyme TLL
Source: PLoS One. 2013 Nov 22;8(11):e80715. doi: 10.1371/journal.pone.0080715 (PMC3838391; doi:10.1371/journal.pone.0080715)
Supplement: Figure S1 — NMR spectra of 6’-ester derivatives of helicid. (DOC) [file pone.0080715.s001.doc]

**Figure S1. NMR spectra of 6’-ester derivatives of helicid.**


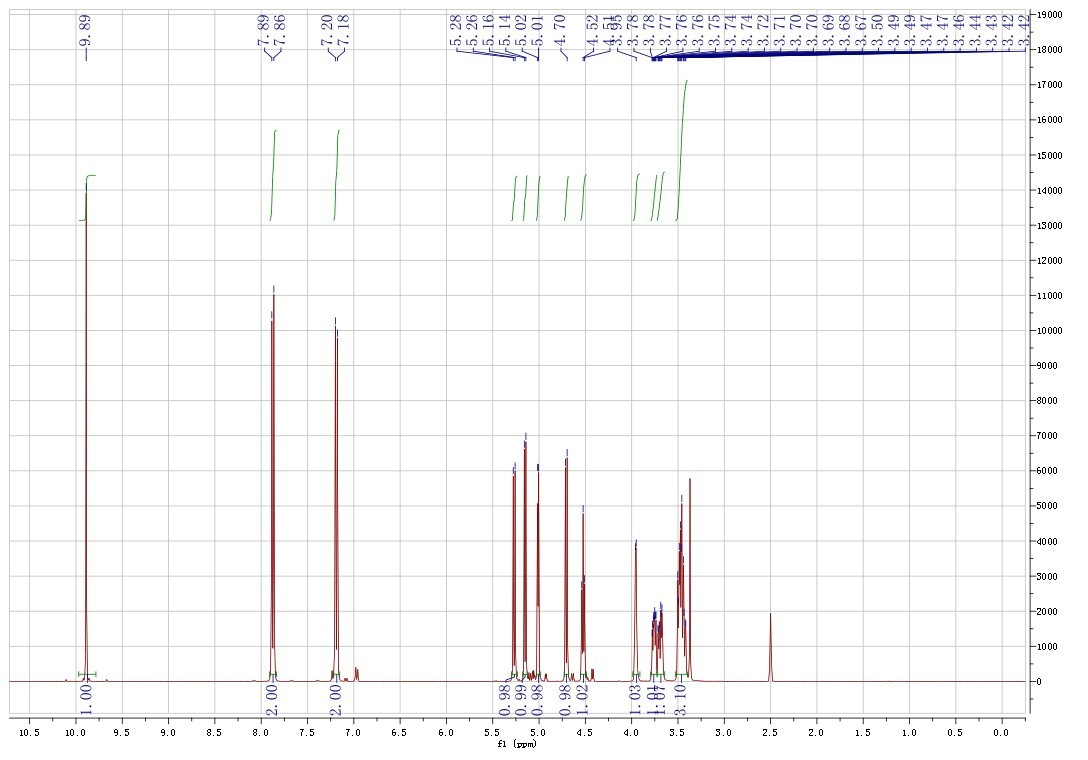


**Helicid (^1^H NMR in DMSO-*d_6_*)**


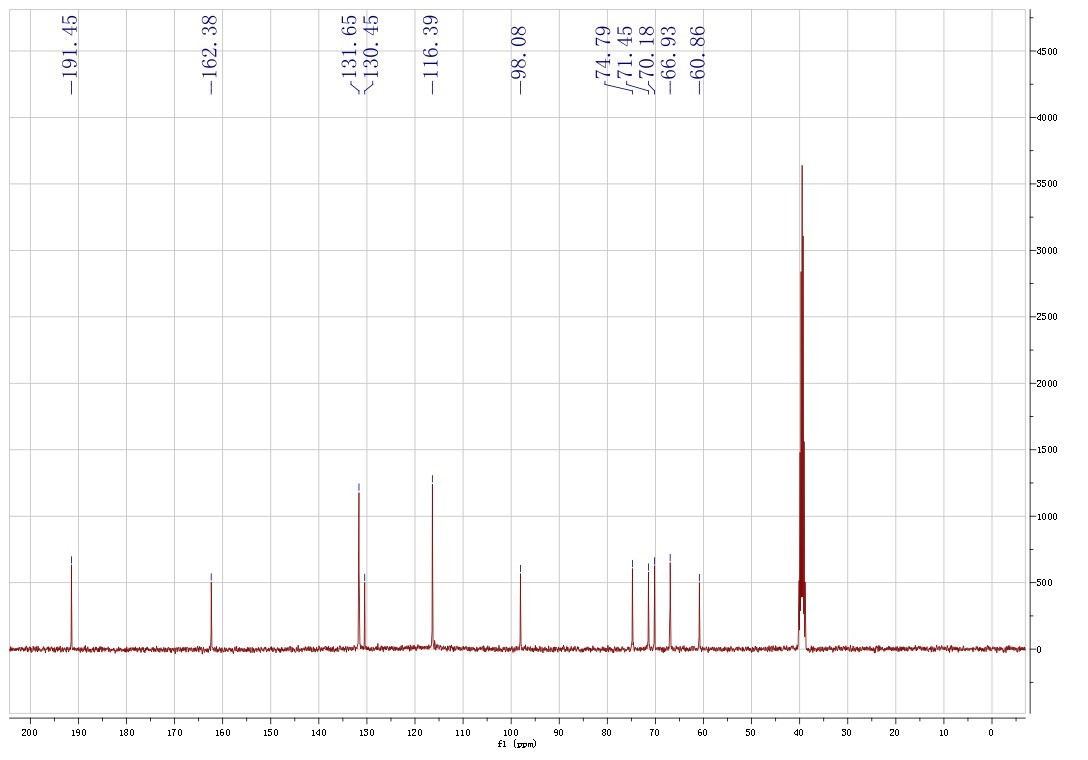


**Helicid (^13^C NMR in DMSO-*d_6_*)**


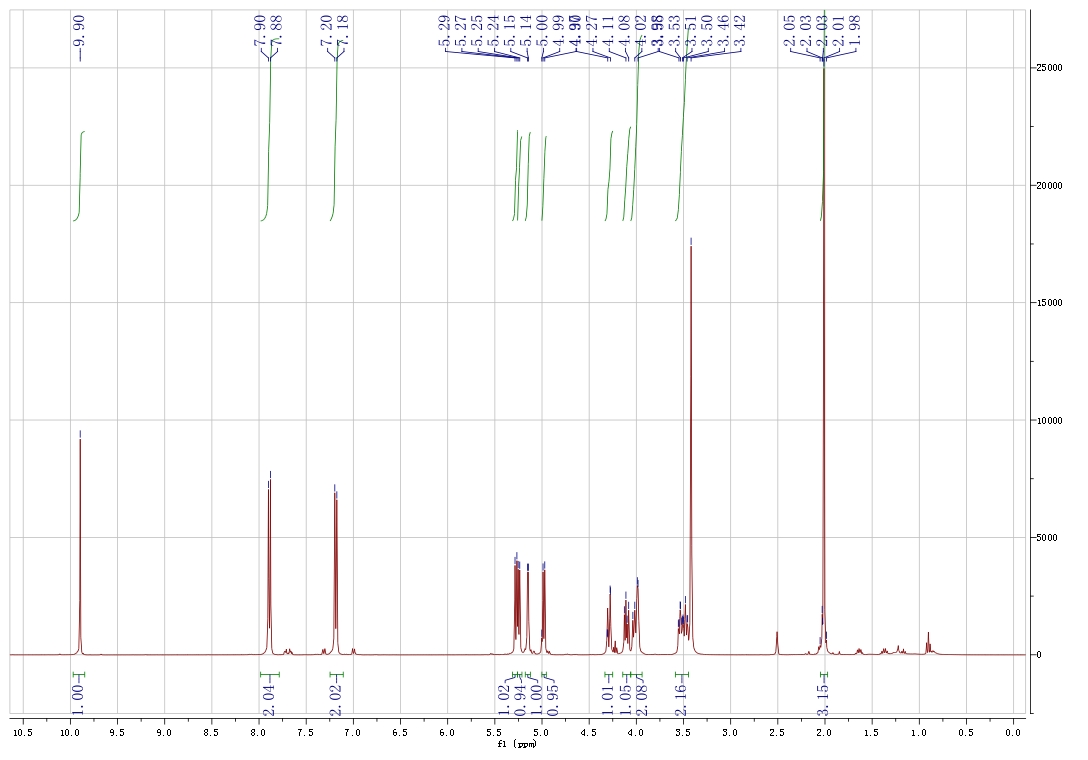


**Helicid 6’-acetate (^1^H NMR in DMSO****-*d_6_*)**


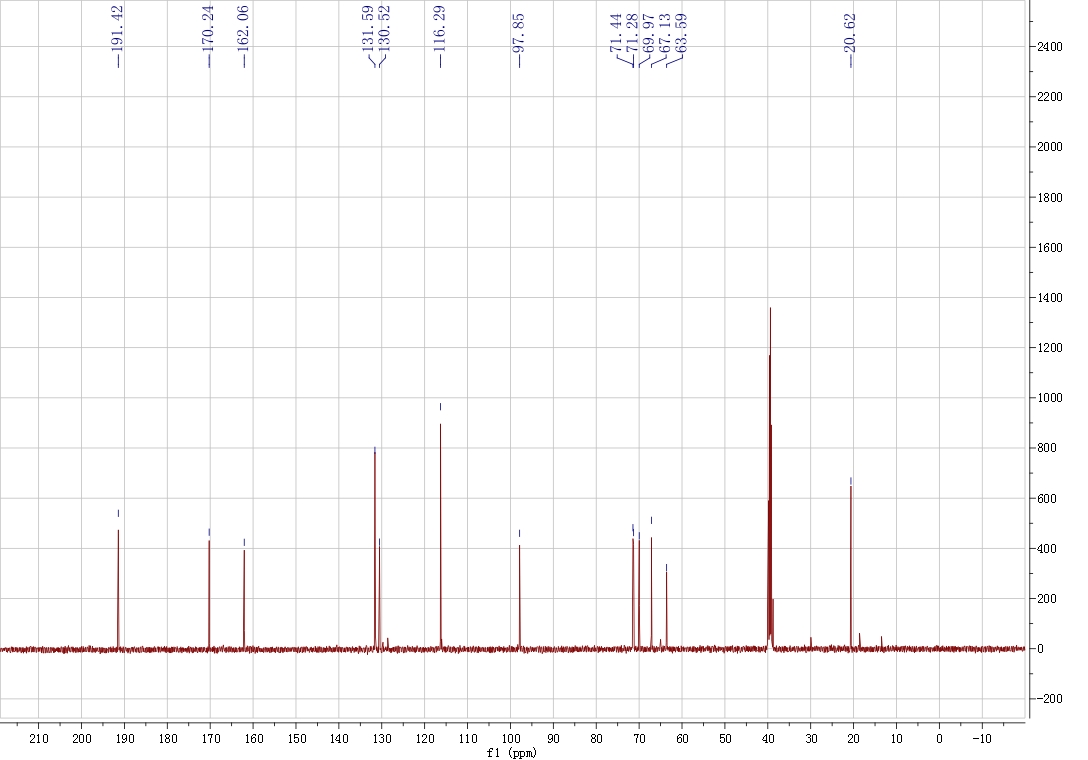


**Helicid 6’-acetate (^13^C NMR in DMSO-*d_6_*)**

**
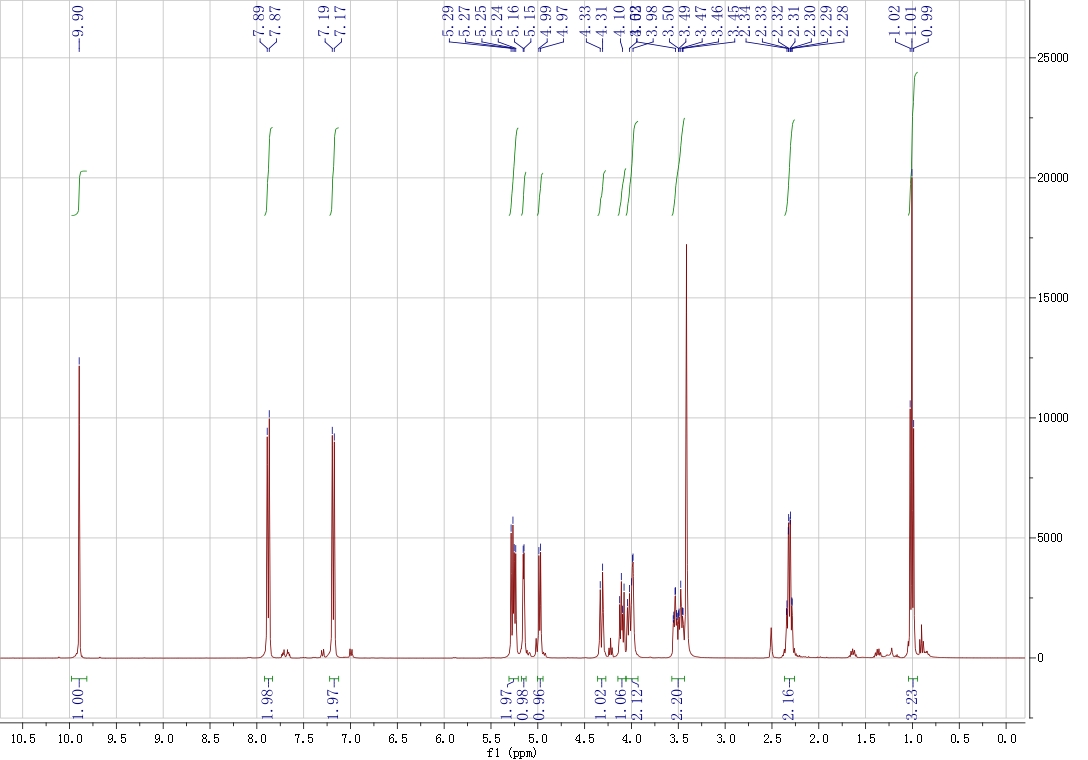
**

**Helicid 6’-propionate (^1^H NMR in DMSO-*d_6_*)**

**
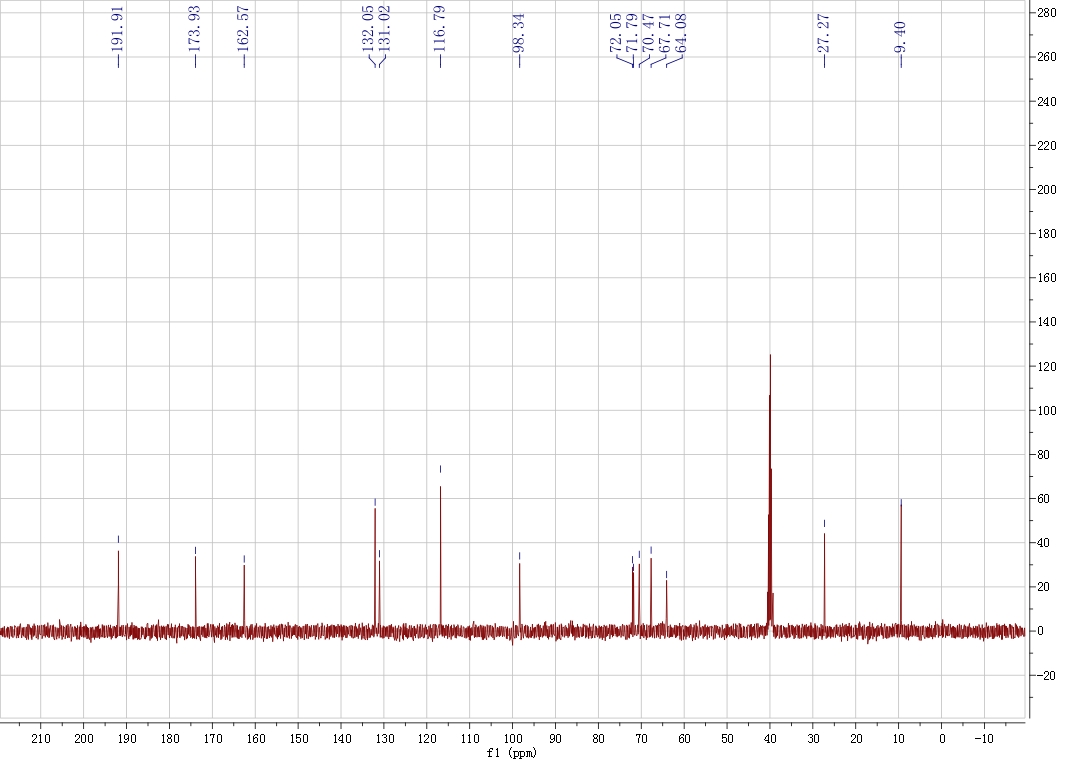
**

**Helicid 6’-propionate (^13^C NMR in DMSO-*d_6_*)**

**
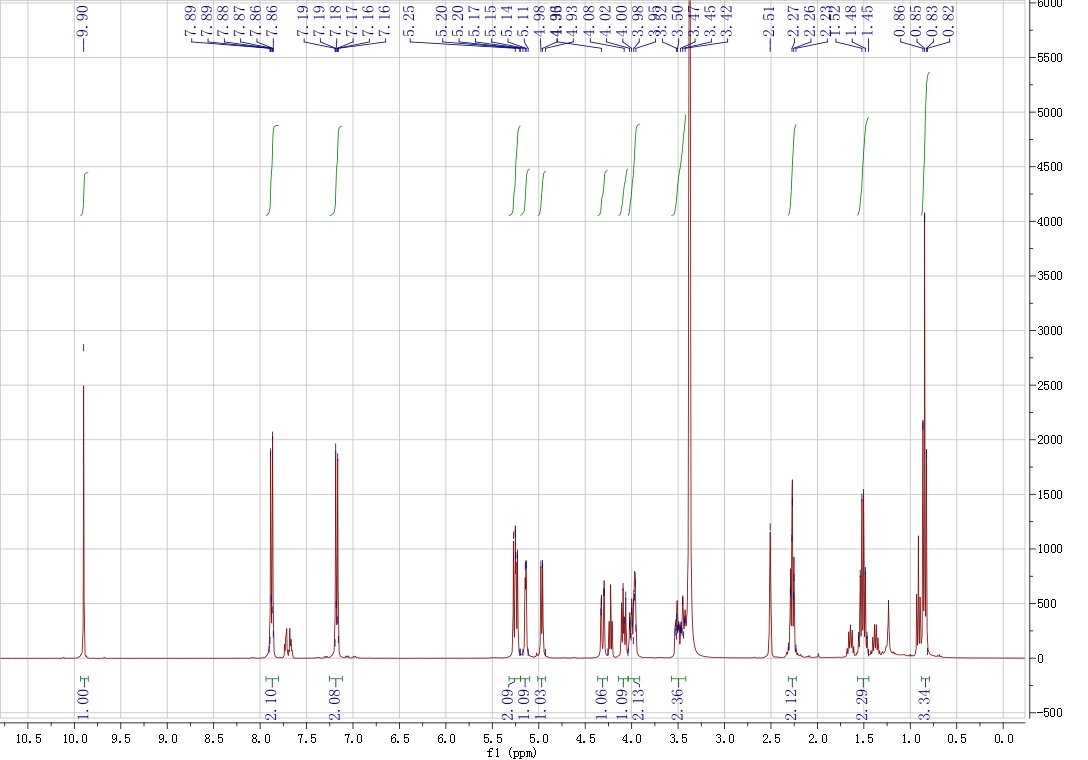
**

**Helicid 6’-butyrate (^1^H NMR in DMSO-*d_6_*)**

**
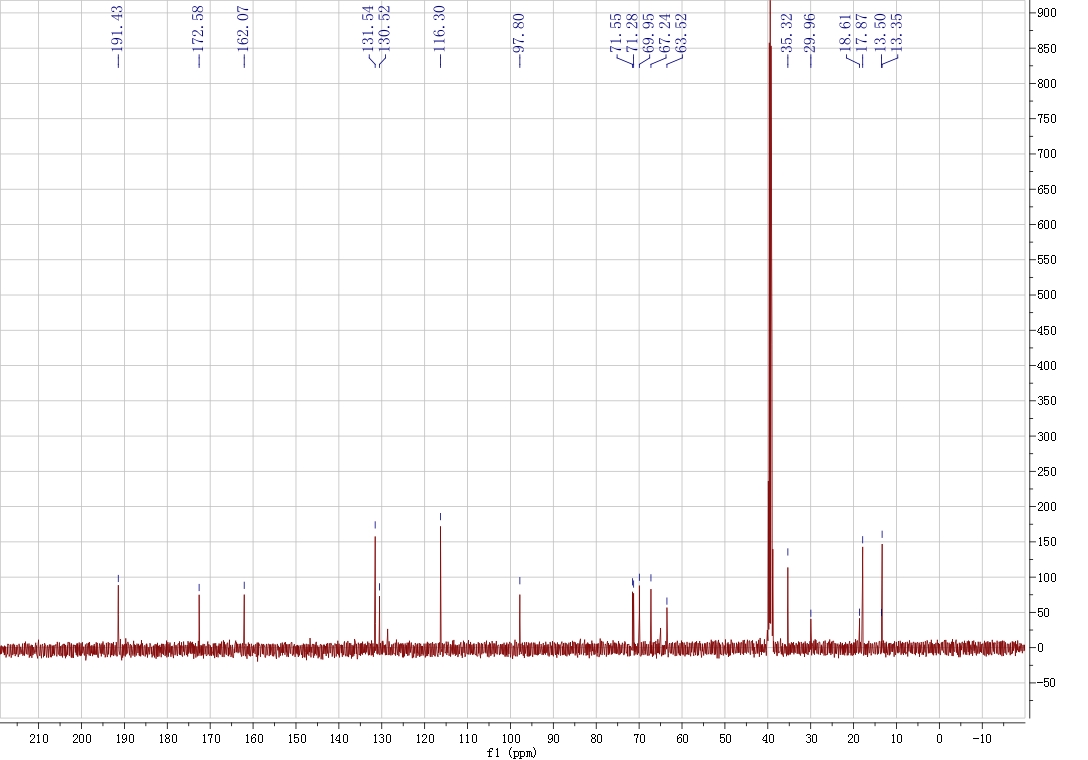
**

**Helicid 6’-butyrate (^13^C NMR in DMSO-*d_6_*)**

**
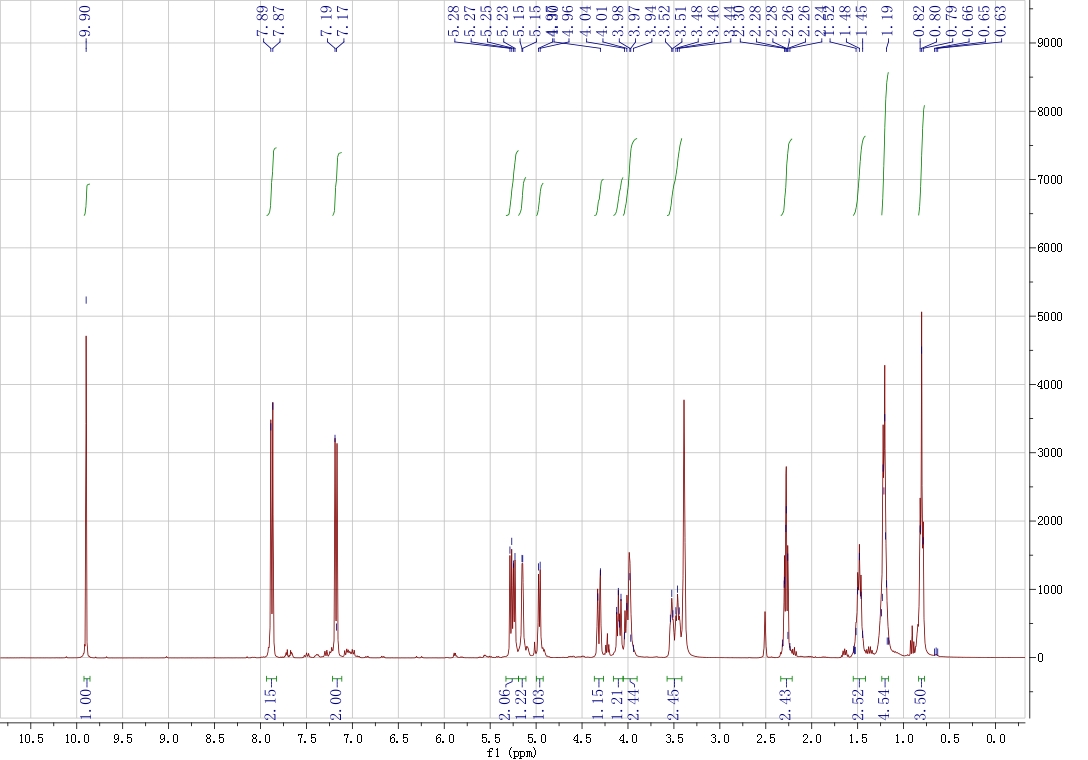
**

**Helicid 6’-hexanoate (^1^H NMR in DMSO-*d_6_*)**

**
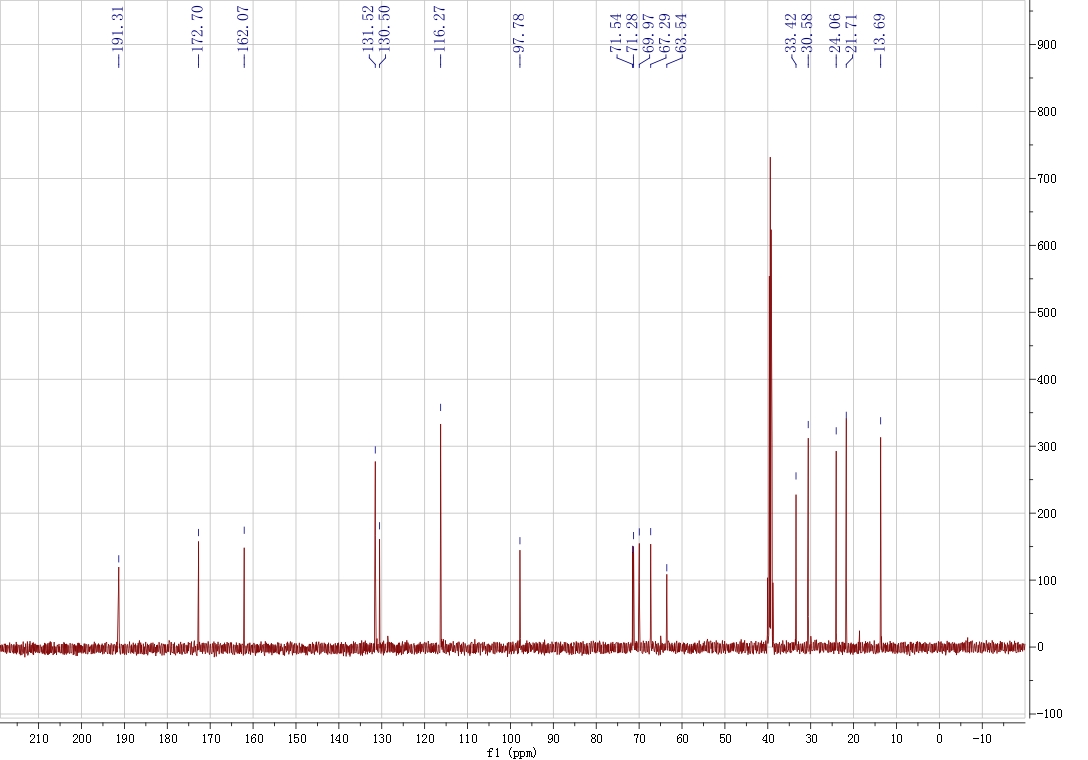
**

**Helicid 6’-hexanoate (^13^C NMR in DMSO-*d_6_*)**

**
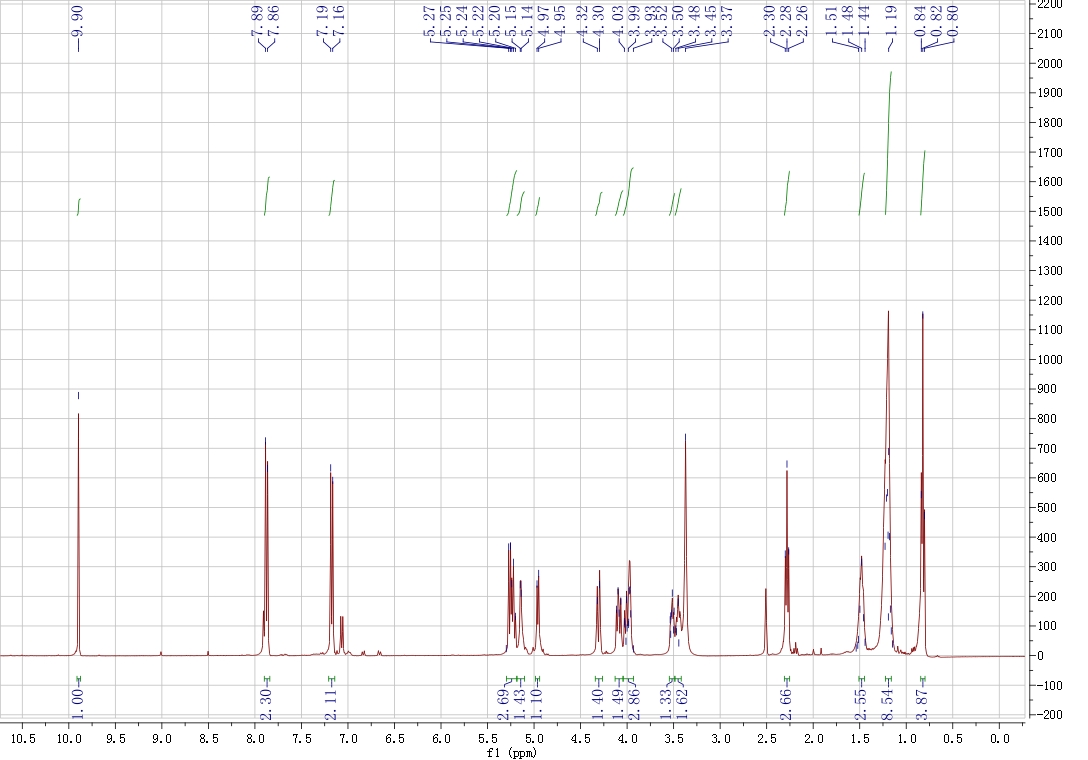
**

**Helicid 6’-caprylate (^1^H NMR in DMSO-*d_6_*)**

**
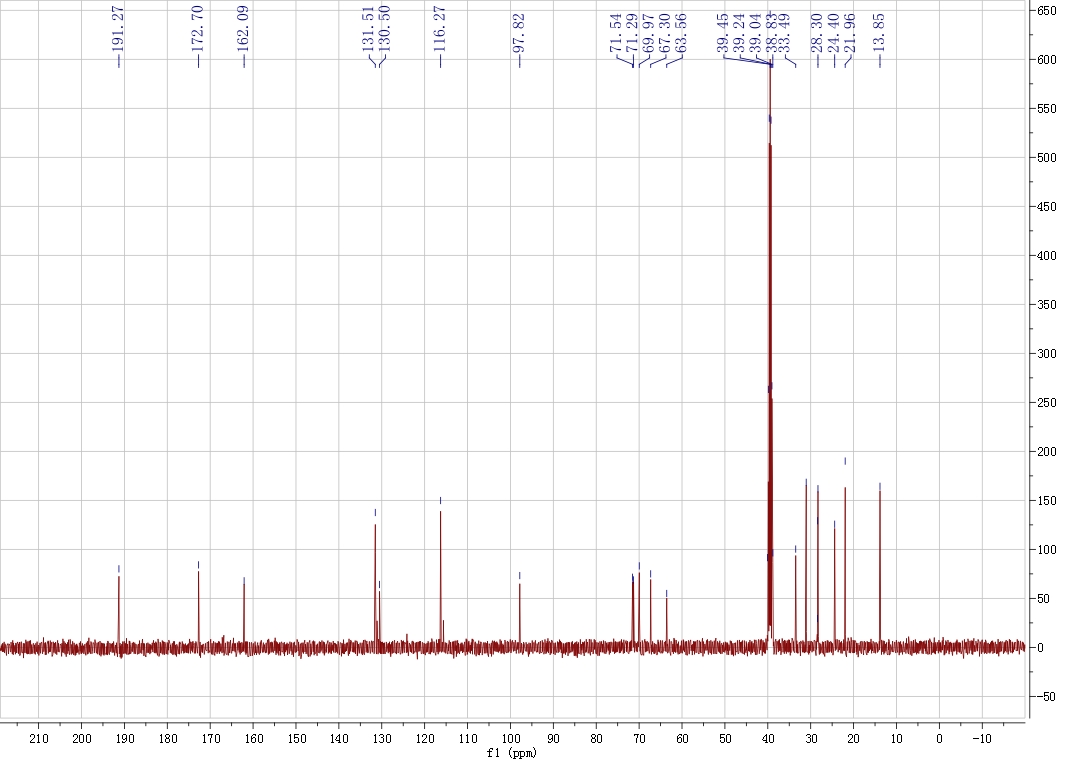
**

**Helicid 6’-caprylate (^13^C NMR in DMSO-*d_6_*)**

**
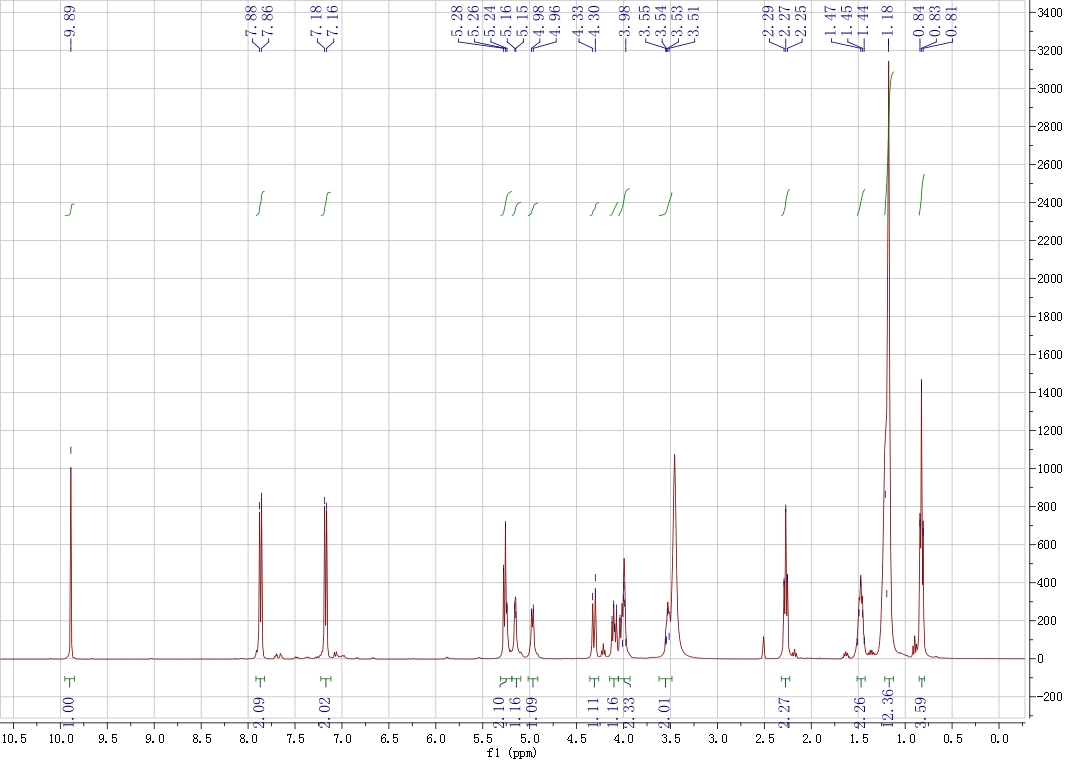
**

**Helicid 6’-decanoate (^1^H NMR in DMSO-*d_6_*)**

**
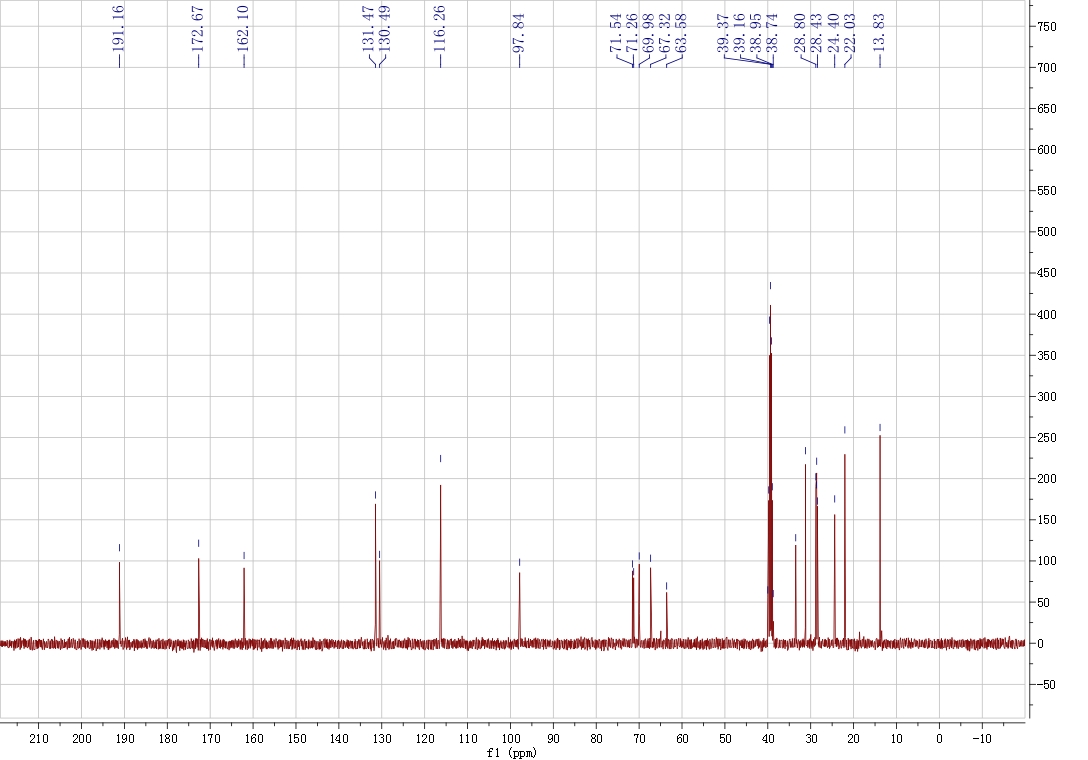
**

**Helicid 6’-decanoate (^13^C NMR in DMSO-*d_6_*)**

**
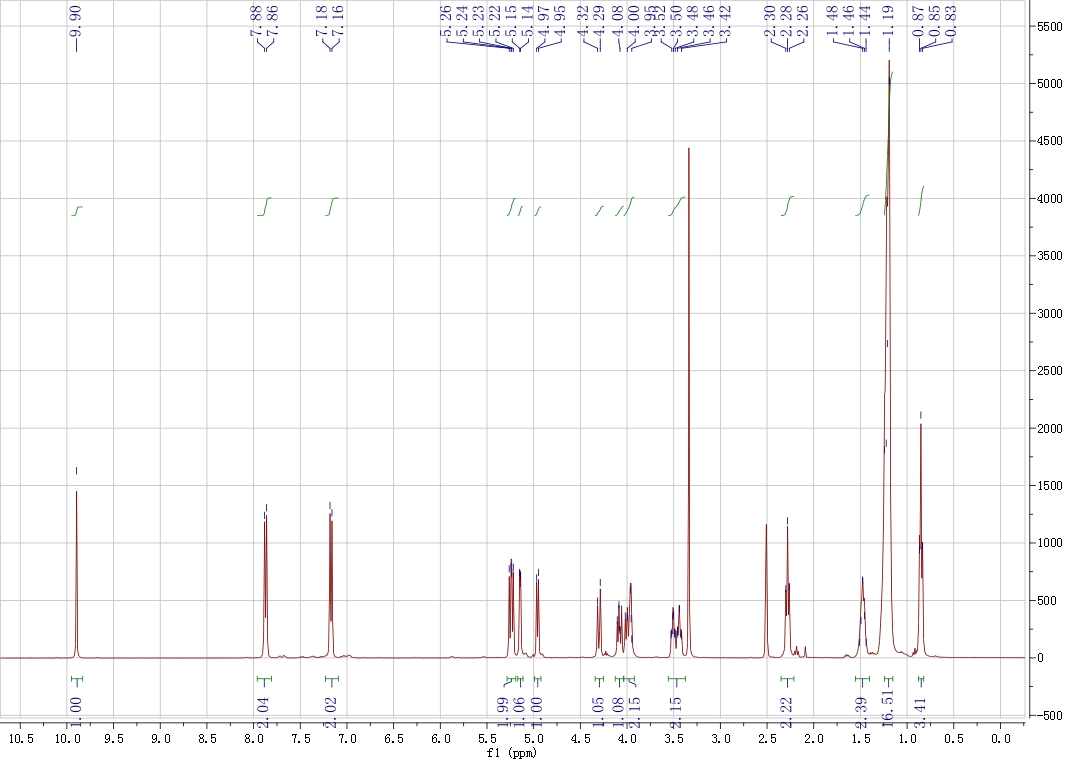
**

**Helicid 6’-laurate (^1^H NMR in DMSO-*d_6_*)**

**
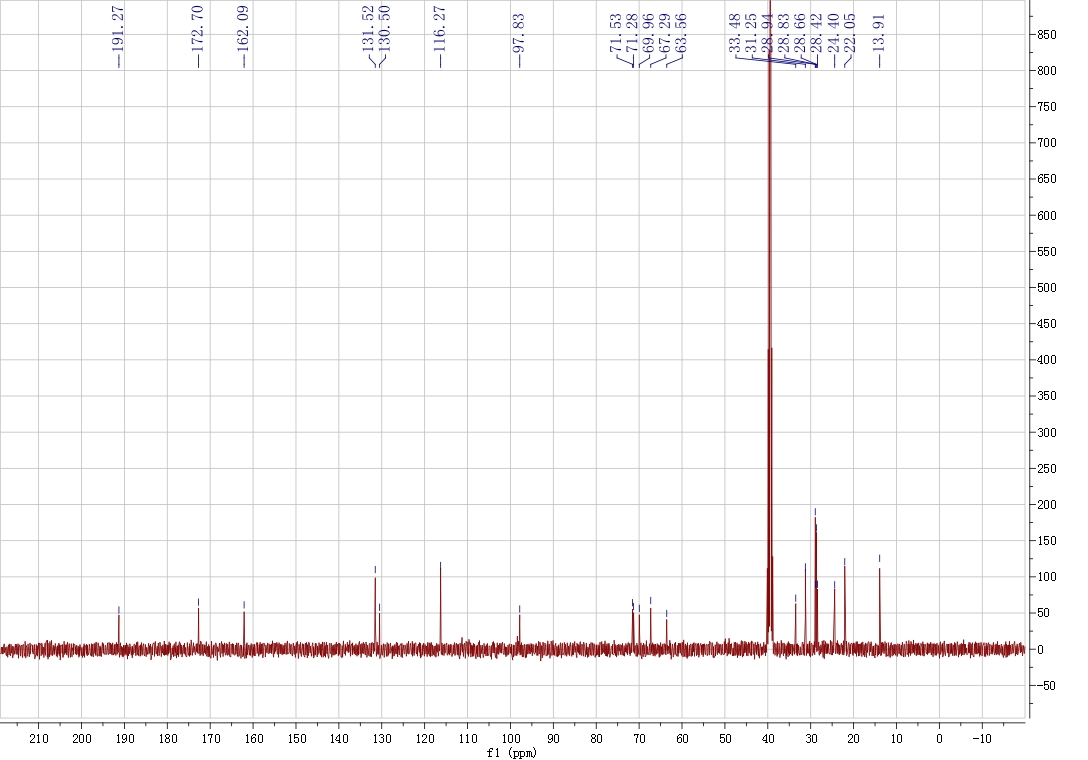
**

**Helicid 6’-laurate (^13^C NMR in DMSO-*d_6_*)**

**
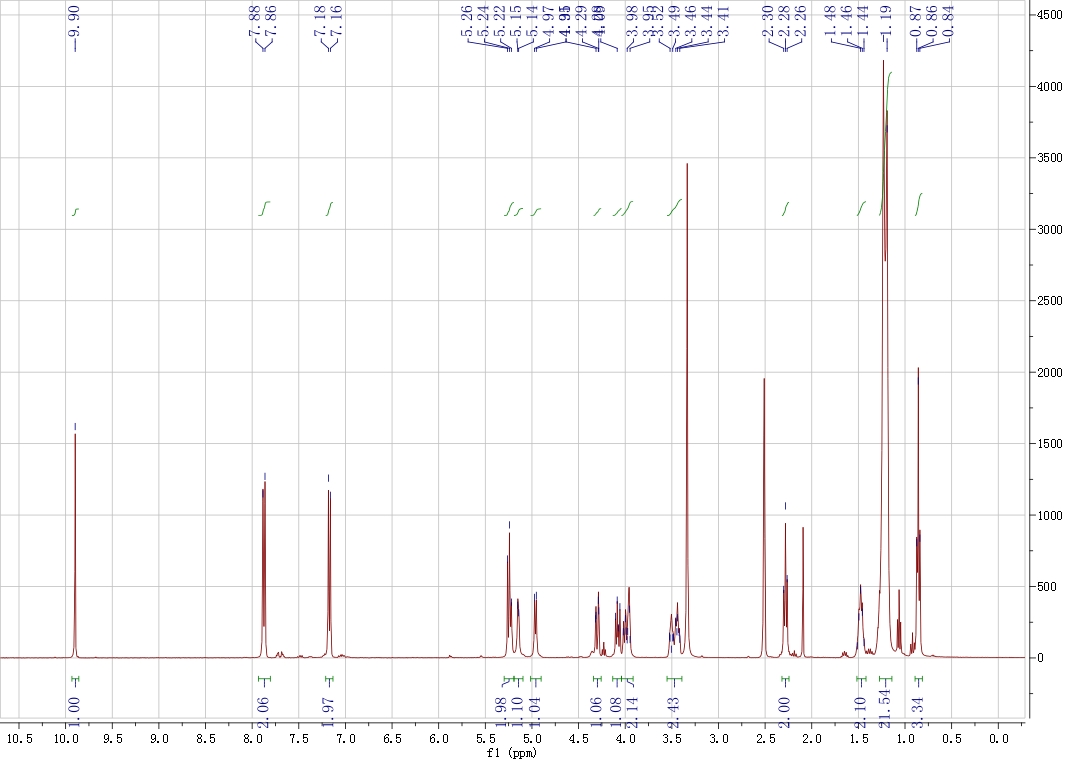
**

**Helicid 6’-myristate (^1^H NMR in DMSO-*d_6_*)**

**
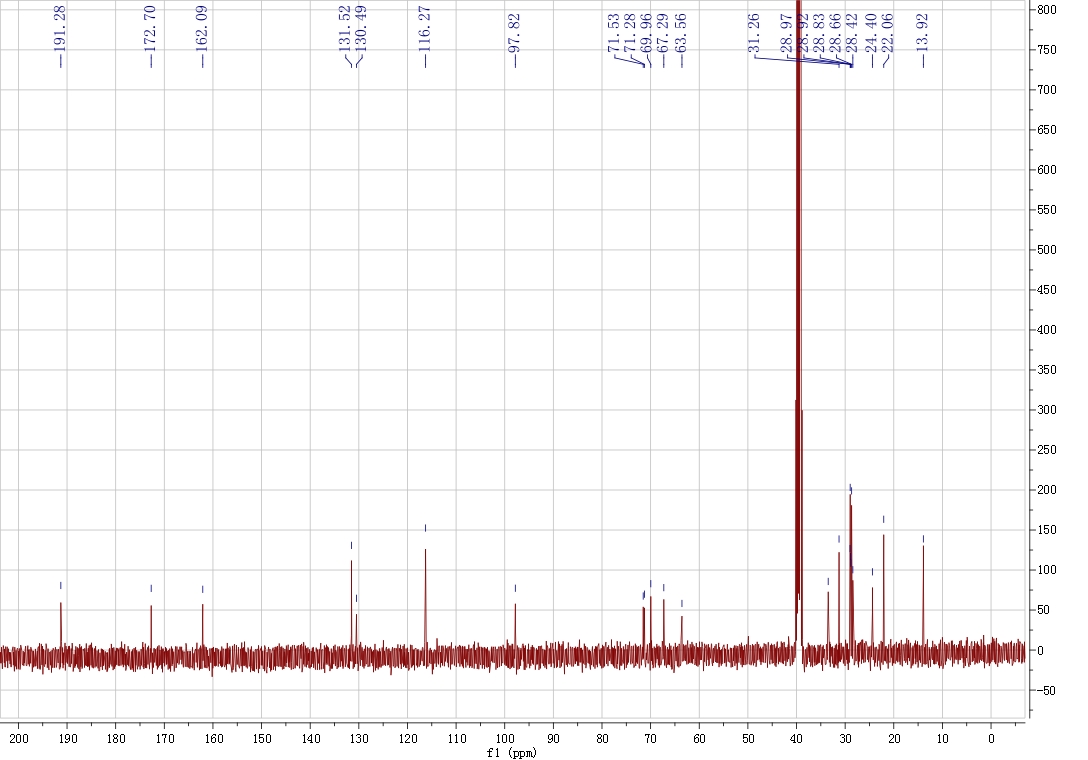
**

**Helicid 6’-myristate (^13^C NMR in DMSO-*d_6_*)**

**
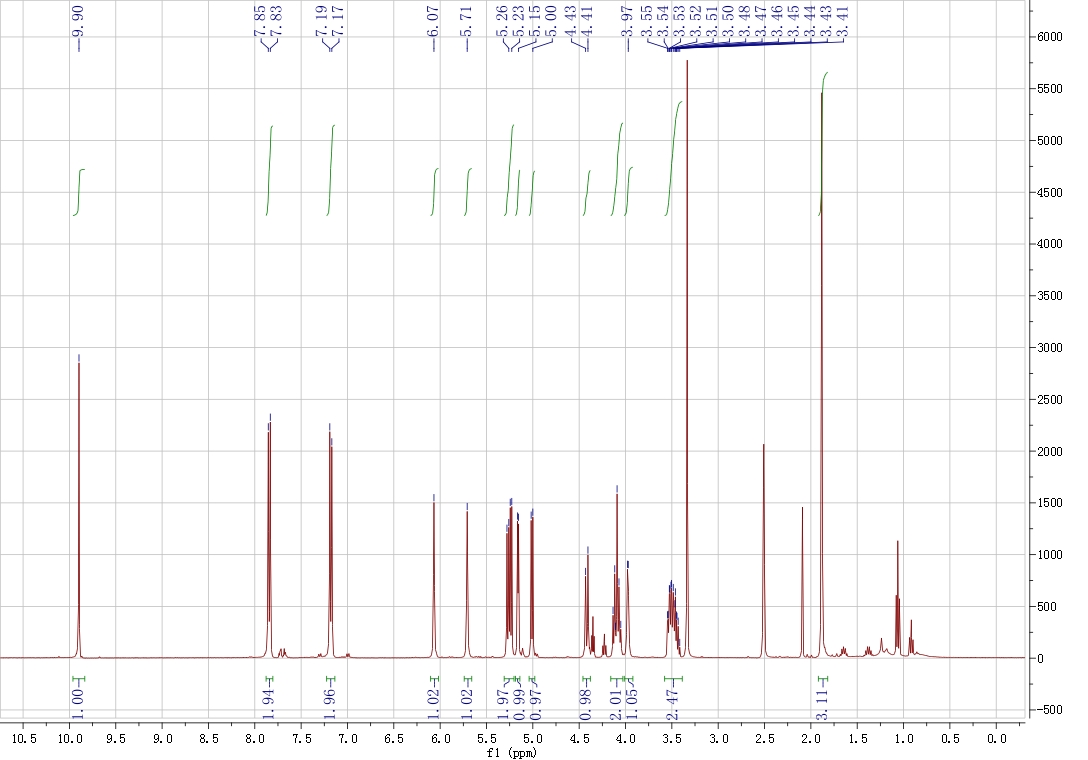
**

**Helicid 6’-methacrylate (^1^H NMR in DMSO-*d_6_*)**

**
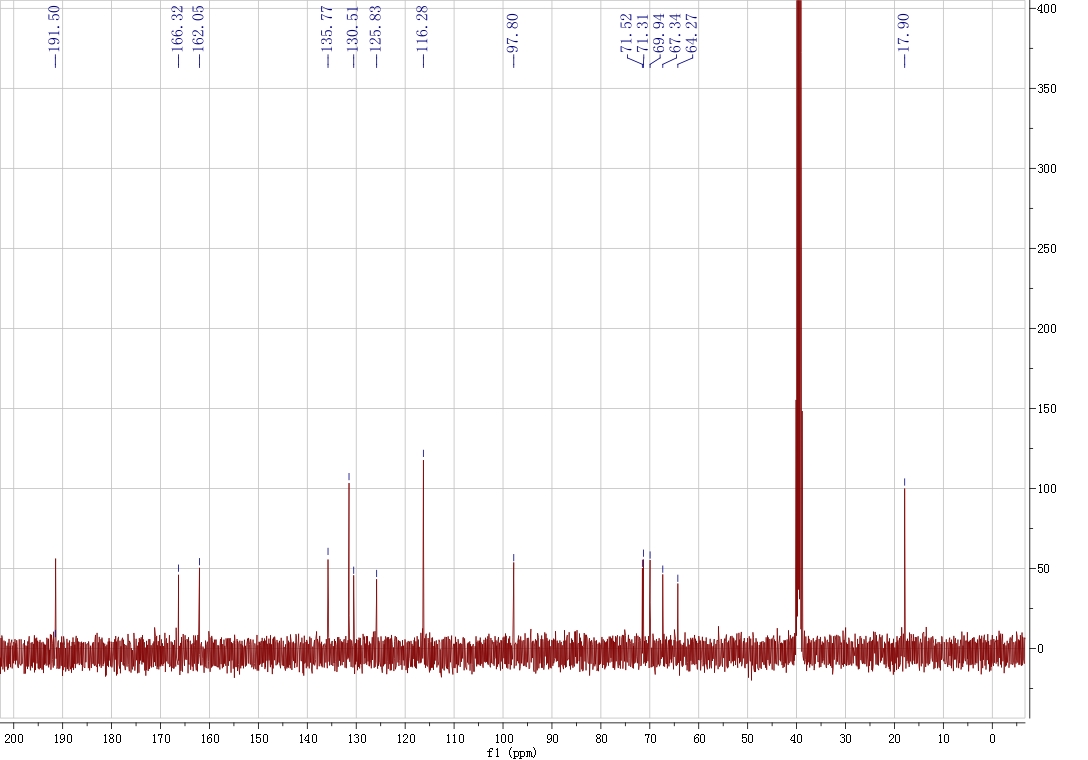
**

**Helicid 6’-methacrylate (^13^C NMR in DMSO-*d_6_*)**

**
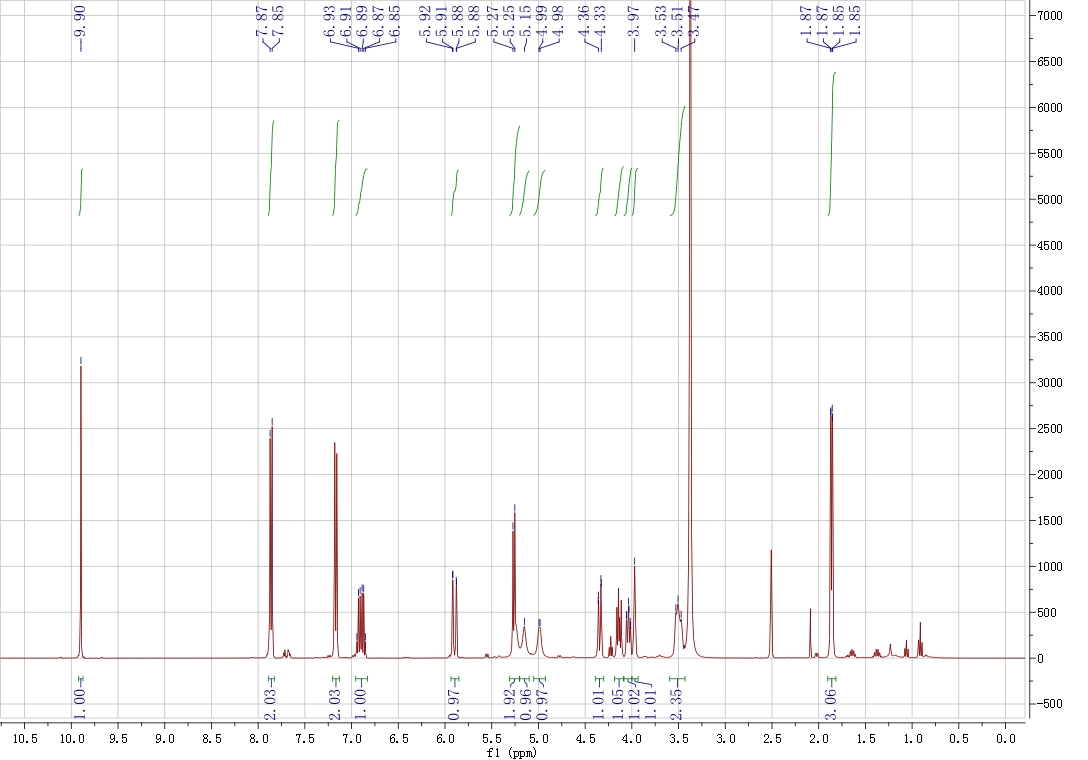
**

**Helicid 6’-crotonate (^1^H NMR in DMSO-*d_6_*)**

**
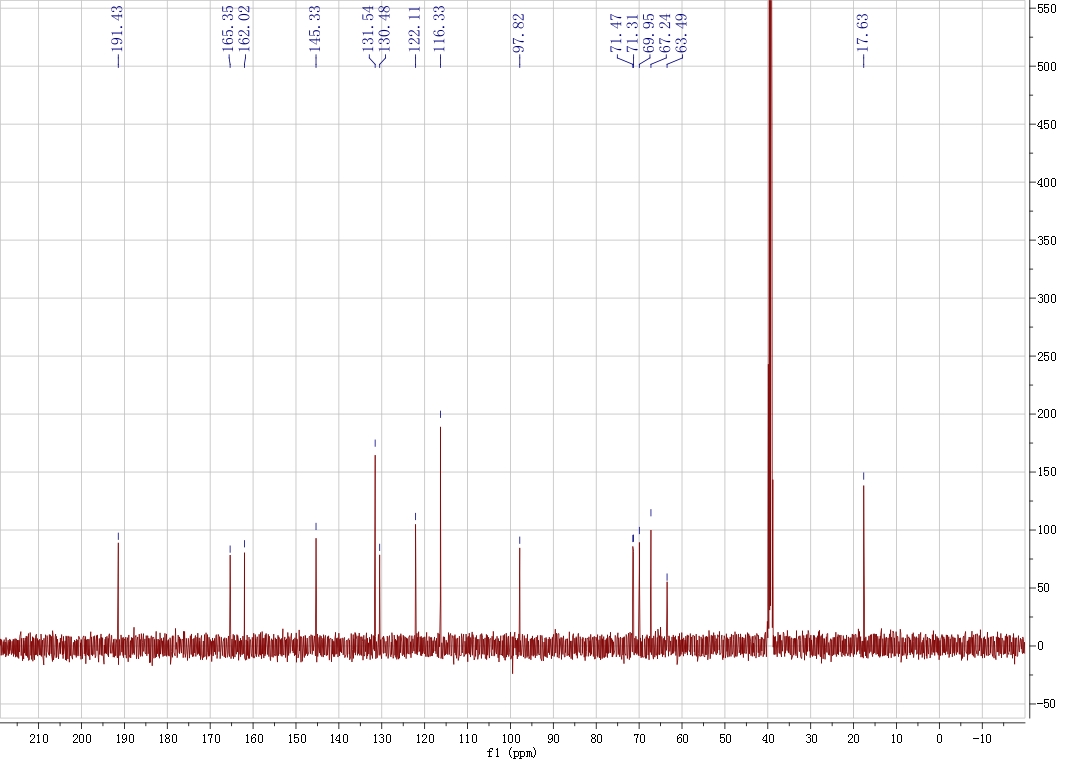
**

**Helicid 6’-crotonate (^13^C NMR in DMS-O-*d_6_*)**
