# Supplementary material for: Novel and Highly Efficient Regioselective Route to Helicid Esters by Lipozyme TLL
Source: PLoS One. 2013 Nov 22;8(11):e80715. doi: 10.1371/journal.pone.0080715 (PMC3838391; doi:10.1371/journal.pone.0080715)
Supplement: Figure S2 — HPLC Chromatograms of 6’-ester derivatives of helicid. (DOC) [file pone.0080715.s002.doc]

**Figure S2. HPLC Chromatograms of 6’-ester derivatives of helicid.**

**Helicid 6’-acetate**

**Helicid 6’-propionate**

**Helicid 6’-butyrate**

**Helicid 6’-hexanoate**

**Helicid 6’-caprylate**

**Helicid 6’-decanoate**

**Helicid 6’-laurate**

**Helicid 6’-myristate**

**Helicid 6’-methacrylate**

**Helicid 6’-crotonate**
