# Supplementary material for: Novel and Highly Efficient Regioselective Route to Helicid Esters by Lipozyme TLL
Source: PLoS One. 2013 Nov 22;8(11):e80715. doi: 10.1371/journal.pone.0080715 (PMC3838391; doi:10.1371/journal.pone.0080715)
Supplement: Figure S3 — MS spectra of 6’-ester derivatives of helicid. (DOC) [file pone.0080715.s003.doc]

**Figure S3. MS spectra of 6’-ester derivatives of helicid.**

**1. Helicid 6’-acetate (C_15_H_18_O_8_)**

MS [ESI(-)] m/z：121([C_7_H_6_O-H]-), 371([M+COOH]-), 651([2M-H]-).

**2. Helicid 6’-propionate (C_16_H_20_O_8_)**

MS [ESI(-)] m/z：121([C_7_H_6_O-H]-), 385([M+COOH]-), 679([2M-H]-).

3. **Helicid 6’-butyrate (C_17_H_22_O_8_)**

MS [ESI(-)] m/z：121([C_7_H_6_O-H]-), 399([M+COOH]-), 707([2M-H]-).

4. **Helicid 6’-hexanoate (C_19_H_26_O_8_)**

MS [ESI(-)] m/z：121([C_7_H_6_O-H]-), 427([M+COOH]-), 763([2M-H]-).

5. **Helicid 6’-caprylate (C_21_H_30_O_8_)**

MS [ESI(-)] m/z：121([C_7_H_6_O-H]-), 455([M+COOH]-), 819([2M-H]-)

6. **Helicid 6’-decanoate (C_23_H_34_O_8_)**

MS [ESI(-)] m/z：121([C_7_H_6_O-H]-), 483([M+COOH]-), 875([2M-H]-).

7. **Helicid 6’-laurate (C_25_H_38_O_8_)**

MS [ESI(-)] m/z：121([C_7_H_6_O-H]-), 511([M+COOH]-), 931([2M-H]-).

8. **Helicid 6’-myristate (C_27_H_42_O_8_)**

MS [ESI(-)] m/z：121([C_7_H_6_O-H]-), 539([M+COOH]-), 987([2M-H]-).

9. **Helicid 6’-methacrylate (C_17_H_20_O_8_)**

MS [ESI(-)] m/z：121([C_7_H_6_O-H]-), 397([M+COOH]-), 703([2M-H]-).

10. **Helicid 6’-crotonate (C_17_H_20_O_8_)**

MS [ESI(-)] m/z：121([C_7_H_6_O-H]-), 397([M+COOH]-), 703([2M-H]-).
